# INTERnational Project for the Evaluation of “activE Rehabilitation” (inter-PEER) – a protocol for a prospective cohort study of community peer-based training programmes for people with spinal cord injury

**DOI:** 10.1186/s12883-019-1546-5

**Published:** 2020-01-11

**Authors:** Anestis Divanoglou, Tomasz Tasiemski, Sophie Jörgensen

**Affiliations:** 1 Department of Physiotherapy, School of Health Sciences, University of Iceland, Stapi v. Hringbraut, IS-101, Reykjavik, Iceland; 2 School of Health, Medical and Applied Sciences, Central Queensland University, Rockhampton, Queensland Australia; 3 Department of Adapted Physical Activity, Poznań University of Physical Education, ul. Królowej Jadwigi 27/39, 61-871 Poznań, Poland; 4 Department of Health Sciences, Rehabilitation Medicine Research Group, Lund University, PO Box 157, SE-221 00 Lund, Sweden; 5 Department of Neurology and Rehabilitation Medicine, Skåne University Hospital, Lund, Sweden

**Keywords:** Community rehabilitation, Active rehabilitation, Spinal cord injuries, Peer mentor, Mentoring, Peer support, Activities of daily living, Self-efficacy, Wheelchair skills

## Abstract

**Background:**

Active Rehabilitation (AR) is a community peer-based concept for people with spinal cord injury (SCI) that is primarily delivered through brief residential training programmes. Despite a plethora of positive anecdotal evidence of AR programmes as life-changing experiences, the effects of AR-programmes have not been evaluated scientifically. Here, we present the protocol of the INTERnational Project for the Evaluation of “activE Rehabilitation” (inter-PEER) aiming to evaluate the effects of AR training programmes on community-dwelling individuals with SCI.

**Methods:**

International prospective cohort study that recruits consecutive participants in AR training programmes. Evaluation is conducted through a web-based survey at 3 time-points: at the commencement and completion of the training programme, and 3 months after the end of the training programme. Evaluation also includes a practical wheelchair skills test at the first two time-points.

The primary outcome measures are the Spinal Cord Independence Measure Self-report (SCIM-SR), the Queensland Evaluation of Wheelchair Skills test (QEWS), the Wheelchair Skills Test Questionnaire (WST-Q) and the Moorong Self-Efficacy Scale (MSES). The secondary outcome measures are the 11-item Life Satisfaction Questionnaire (LiSat-11), the Utrecht Scale for Evaluation of Rehabilitation-Participation (USER-Participation), the Leisure Time Physical Activity Questionnaire for people with SCI (LTPAQ-SCI) and the 10-item Connor-Davidson Resilience Scale (CD-RISC-10).

We piloted the implementation of the protocol in Sweden in 7 participants with diverse SCI and sociodemographic characteristics and collected feedback from participants and peer-mentors about study procedures through interviews, a workshop and field observations.

**Discussion:**

Inter-PEER is the first initiative to propose a systematic evaluation of the effects of AR training programmes among individuals with SCI. The project is a collaborative work of multiple stakeholders, including researchers, clinicians, peer mentors with SCI, and administrators of organisations providing AR programmes. The inter-PEER uses standardised outcome measures relevant to the AR context, it will facilitate quality evaluations of community peer-based programmes, stimulate international collaborations, and inform the design of randomised controlled trials on the effects of AR training programmes.

## Background

Despite access to specialised acute care and in-patient rehabilitation, newly injured individuals with spinal cord injury (SCI) often feel unprepared physically and psychologically to transition to home [1, 2]. High functional independence, high everyday social support and high self-efficacy have been reported as being key determinants of life satisfaction during the first five years after SCI [3]. Because of the long and often complex adjustment process, it has been recommended that services, information and resources facilitating the adjustment process are offered multiple times and in multiple ways [4]. Community organisations play a key role in the post-discharge life of community-dwelling individuals with SCI by providing such ongoing support services [5].

Active Rehabilitation (AR) is a community peer-based concept for people with spinal cord injury (SCI) that was developed in Sweden 40 years ago. Since then, aspects of the concept have been implemented in more than 20 countries worldwide [5]. The objective of AR is to promote healthy active living by providing ongoing support, education and skills training based on peer interaction [5]. The concept is primarily delivered through brief residential training programmes that provide group-based yet individualised training opportunities in a community-like environment [5]. The 10 key elements of AR were described recently and despite some country-specific adaptations, they were present in programmes offered in 21 countries in Europe, Asia and Africa [5]. We also found that the community organisation “RG AR” from Sweden had facilitated the development of the concept in five of these countries, where the concept was implemented in a very similar way as in Sweden [5].

Anecdotally, participants with SCI have described AR programmes as a life-changing experience, but scientific evidence of the effects of such programmes is lacking. Recent systematic and scoping reviews identified very few scientific publications evaluating community peer-based programmes for people with SCI, and none evaluating outcomes of AR programmes [6, 7]. This apparent lack of evidence might have negative consequences for the referral processes from healthcare providers, funding, availability and development of the programmes. The lack of published research investigating AR could be attributed to its core characteristics, i.e. consumer driven, practice-oriented with a strong emphasis on service delivery rather than on research. It could also be explained by the AR community organisations lacking the skills and knowledge required to undertake research, their limited resources and their reliance on volunteers [6]. Recently, a number of organisations offering AR services partnered with researchers to establish and implement outcome evaluation processes in the context of AR. The protocol described in this paper is the first result of this partnership.

The current paper presents the protocol of the INTERnational Project for the Evaluation of activE Rehabilitation (inter-PEER), which is the first comprehensive scientific evaluation of the AR training programmes for participants with SCI. The primary objective of inter-PEER is to measure the effects of AR training programmes on physical independence, self-efficacy and wheelchair skills among individuals with SCI. The secondary objective is to measure the effects on community participation, life satisfaction, level of physical activity and resilience. The purpose of this protocol is to present the methodology of inter-PEER to facilitate the utilisation of homogenous research methods across organisations that use aspects of the AR programmes, and to facilitate comprehensive programme evaluation and international pooling of data. The inter-PEER protocol could be used as a whole or in part to evaluate AR programmes across countries.

## Methods/design

### Study design

Inter-PEER is an international prospective cohort study that has commenced in Sweden and will later be implemented in other European countries. It involves participants with SCI taking part in AR training programmes that last for 7 days or more. Participant evaluation takes place at 3 time-points: at the commencement (baseline) and completion of the training programme, and 3 months after the end of the training programme. The reporting for studies following this protocol will be guided by the Strengthening the Reporting of Observational studies in Epidemiology (STROBE) statement for cohort studies [8]. Additional file 1 describes the intervention provided in AR programmes. The description of AR programmes adheres to the Template for Intervention Description and Replication (TiDieR) guidelines [9], and it is informed by the Rehabilitation Treatment Specification System [10] and the Spinal Cord Injury Interventions Classification System [11]. A pilot phase of the protocol took place in Sweden between November 2017 and February 2018. Participant recruitment in Sweden began in April 2018.

### Inclusion criteria

All participants in AR training programmes are invited to participate in the study, if they meet the following inclusion criteria: (1) having a SCI (acquired traumatic and non-traumatic, and congenital, e.g., spina bifida); (2) being 16 years or older; (3) being able to comprehend and answer written questions. From a technical point of view, the inter-PEER protocol can only be implemented in AR programmes where there is availability of laptops or tablets with Internet access.

### Procedures

Before the training programme, all registered programme participants receive an e-mail with information about the programme evaluation, emphasizing that participation is voluntary. On the first day of the programme, the on-site data collection coordinator approaches participants as they arrive, provides an information letter, offers to provide more information about the programme evaluation and then asks participants to provide a written informed consent. The on-site data collection coordinator has some research experience and has received training in the data collection procedures of Inter-PEER. An online platform, such as Survey Monkey, is used to collect data. For the start and completion of the programmes, laptops or tablets with Wifi or cellular connection are provided to participants to complete the survey. For the 3-month follow-up, participants are provided with an individualised link that they use to complete the survey in their own time and their own laptop or tablet device.

During the first 24 h of the AR programme, consenting participants complete the online survey and also conduct a practical wheelchair skills assessment that is administered by peer mentors. A similar process takes place on completion of the programme. At the end of the programme, the on-site data collection coordinator completes a form with the fidelity criteria for the specific programme. Three months later, participants are provided with an individualised link and are asked to complete the follow-up online survey. Participants complete the 3-month follow-up evaluation in their own time online. Because participants often live far away from the training programme location, the practical wheelchair skills test is not included in the 3-month follow-up evaluation for practical reasons.

The 3-month period after completion of an AR training programme was chosen as the optimal time for final evaluation. We believe that a shorter period would be insufficient to see potential progress after the AR training. On the other hand, a longer period (e.g. 6 or 12 months) may increase drop-outs and introduce confounding factors which would be difficult to control for given that the study does not have a control group. At 3-month follow-up, participants are asked to indicate whether any new training (other than the AR programme) and any other events (e.g. surgeries, hospitalisations) may have positively or negatively impacted their status. Respondents have the opportunity to explain further through free text.

The inter-PEER has a strong focus on embedding the outcome evaluation in the schedule of AR programmes. Better integration of the evaluation in the schedule and greater engagement of peer mentors and non-disabled members in the evaluation processes can promote sustainability, increase participation and retention rates, and improve quality of data.

If sufficient funding and resources are available, organisations can consider employing a multiple baseline design, for example by including an additional baseline assessment several weeks prior to start of the programme. In that case, it is recommended that the primary outcome measures are administered rather than the full survey. The latter would improve the design of the study and would provide more confidence in inferring causation as a result of the programme.

Each country has their own data collection and steering team and can decide if they provide data for pooling. Organisations may choose to adopt and implement the full protocol and battery of outcome measures, or may choose a part of it based on their resources, needs and context.

### Data collection

The full list of outcome measures and the scheme for administering each one of them are presented in Table 1. All outcome measures are self-reported except the Queensland Evaluation of Wheelchair Skills test (QEWS) that is a skill-based evaluation.

**Table 1:** Outcome measures at the INTERnational Project for the Evaluation of activE Rehabilitation (inter-PEER)

Domain	Outcome measure	Number of items	Score range	Evaluation points		
				T1	T2	T3
*Sociodemographic and injury-related factors*						
Demographic and lesion characteristics	Gender, date of birth, current marital status, number of individuals living in the household, need for assistance with day-to-day activities and who provides this, highest level of education competed, household income, level of lesion, completeness of lesion, cause of injury.^a^ Number of previously attended AR training programmes.	17		√		
Adverse events during the last 3 months	Respondents are asked to select all relevant options: A fall that resulted in a fracture, a sprain or something similar; a skin injury due to fall or other activity (e.g. transfer, outdoor activity); a fall that did not result in an injury; more frequently occurring urinary tract infections than usual; prolonged fatigue; a pressure ulcer; emotional breakdown; increased fear with performing activities (e.g. transfers, sports); increased concern for the future in relation to spinal cord injury.	1		√	√^b^	√
*Primary outcome measures*						
Functional level	Spinal Cord Independence Measure Self-report (SCIM-SR) [﻿12﻿]	17	0–100	√	√	√
Practical wheelchair skills	Queensland Evaluation of Wheelchair Skills (QEWS) [﻿13﻿]	5	0–25	√	√	
Self-reported wheelchair skills	Wheelchair Skills Test Questionnaire (WST-Q) [﻿14﻿]	24 (Only items 10, 12–34)	0–100	√	√	√
Self-efficacy	Moorong Self-efficacy Scale (MSES) [﻿15﻿]	16	16–112	√	√	√
*Secondary outcome measures*						
Life satisfaction	Life Satisfaction Questionnaire 11 (LiSat-11) [﻿16﻿]	11	11–66	√		√
Participation	Utrecht Scale for Evaluation of Rehabilitation-Participation (USER-Participation) [﻿17﻿]	22 (Only Frequency and Restriction domains)	0–100 for each domain	√		√
Physical Activity	Leisure Time Physical Activity Questionnaire for people with SCI (LTPAQ-SCI) [﻿18﻿]	2 (Only moderate and vigorous physical activity)		√	√^b^	√
Resilience	Connor-Davidson Resilience Scale 10 (CD-RISC 10) [﻿19﻿]	10	0–100	√	√^b^	√

T1: Commencement of training programme

T2: Completion of training programme

T3: 3 months after completion of the training programme

^a^ Adapted from the International Spinal Cord Injury Survey (InSCI) [20]

^b^ During the last 7 days (duration of the training programme

#### Sociodemographic and injury-related factors

Seventeen questions about sociodemographic and injury related factors are included in the survey. The phrasing of the questions has been adapted from that used in the International Spinal Cord Injury (InSCI) Community Survey [20]. Adverse events are reported at all three time-points of data collection. Based on feedback from stakeholders and on personal experience of the researchers, we developed a list of adverse events that comprises a list of 9 options. Table 1 provides more details for these variables.

#### Primary outcome measures

Based on the focus and content of the AR programme, the Spinal Cord Independence Measure self-report (SCIM-SR), the QEWS, the Wheelchair Skills Test Questionnaire (WST-Q) and the Moorong Self-efficacy Scale (MSES) are the primary outcome measures.

- Functional level is assessed through the SCIM-SR [﻿12﻿]. SCIM is the most widely used outcome measure to assess the physical independence in people with SCI. Its self-report version has shown good validity and reliability [﻿12﻿]. It comprises 17 items divided into three sections: a. self-care; b. respiratory and sphincter control and c. mobility (indoor and outdoor). Each item has a weighted score in relation to the subjective value of the activity, the level of difficulty when performing the task, and the time required [21]. Scores range between 0 and 100 with higher scores indicating a higher functional level.
- The QEWS is used to measure wheelchair skills. QEWS was initially designed for use in people with SCI in the acute hospital setting as well as in the community without extensive or specialized testing equipment [13]. It is short (5 items), simple (easy for peer mentors to administer), and suitable for the AR context (can be easily integrated into the schedule of the programme). Gollan et al. [13] reported that QEWS is reliable, valid and sufficiently sensitive to detect change over a 10-week period of in-patient rehabilitation.
- The WST-Q version 4.3 for manual wheelchairs operated by their users is a self-reported survey measuring capacity (“Can you do it?”), confidence (“How confident are you?”), performance (“How often do you do it?”) and goal attainment (“Is this a training goal?”) for a list of wheelchair skills [14]. The WST-Q has shown good content, construct and concurrent validity for individuals with SCI [22] and high correlation with the observer rated version of the scale [23]. We include 24 out of 34 available skills and only the questions relevant to capacity and confidence [﻿14﻿]. The total sum for each domain (i.e. capacity and confidence) is converted to a score from 0 to 100 based on the number of valid answers, with a higher score representing higher capacity and confidence.
- Self-efficacy is assessed through the MSES [﻿15﻿]. The MSES is a 16-item scale rating confidence in the ability to control behaviour and outcomes on a 7-point Likert scale (1 = very uncertain, 7 = very certain) [﻿24﻿]. It was developed specifically for people with SCI, and a highly rated MSES has been correlated with superior health-related outcomes [24]. It consists of three factors: personal function self-efficacy (e.g. I can maintain my personal hygiene with or without help), social functioning self-efficacy (e.g. I can enjoy spending time with my friends), and a general self-efficacy (e.g. I can accomplish most things I set out to do) [24]. The MSES has shown strong evidence of construct validity, stability and internal consistency [15, 24]. The total score is the sum of all answered items with higher scores indicating high self-efficacy or stronger beliefs in the person’s ability to control their behaviour and outcomes [15].

#### Secondary outcome measures

- Life satisfaction is assessed through the Life Satisfaction Questionnaire-11 (LiSat-11) [﻿16﻿]. This widely used survey has been used across diverse types of conditions. It comprises 11 questions that cover global satisfaction with life (1 item) and domain-specific life satisfaction in 10 items: vocational, financial and leisure situations, contacts with friends, sexual life, self-care management, family life, partner relationships, physical and psychological health. Each item is scored on a 6-point scale from 1 (very dissatisfied) to 6 (very satisfied). Higher scores indicate higher level of life satisfaction. The LiSat-11 is valid for the general population [16] and has shown satisfactory internal consistency in people with SCI [25].
- Participation is assessed through the Utrecht Scale for Evaluation of Rehabilitation participation (USER-Participation) [17]. This International Classification of Functioning Disability and Health (ICF)-based participation assessment includes 32 items that are divided into three domains: (1) frequency of participation, (2) restrictions of participation, and (3) satisfaction with participation. The total sum for each domain is converted to a score from 0 to 100, with a higher score representing higher frequency, less restrictions and higher satisfaction. USER-Participation has been validated in a heterogeneous sample of rehabilitation outpatients [26]. To reduce overlap with the LiSat-11, we have excluded the satisfaction section of USER-Participation.
- Participation in moderate and vigorous physical activity is assessed through the Leisure Time Physical Activity Questionnaire for people with SCI (LTPAQ-SCI) [18]. This is an SCI-specific, self-report measure of leisure time physical activity (LTPA) (i.e. physical activity performed during free time, such as exercising, wheeling and recreational activities) [27]; that assesses minutes of mild, moderate, and heavy intensity LTPA performed over the previous 7 days. Based on the requirement to reach at least moderate intensity LTPA to achieve health benefits [28] and on feedback from respondents during the pilot, we decided not to collect data on mild LTPA.
- Resilience is assessed through the 10-item Connor-Davidson Resilience Scale (CD-RISC 10) [19, 29]. This brief version of CD-RISC comprises 10 items that assess the self-reported ability to cope with adversity [19, 29]. Respondents rate each item on a five-point Likert scale from 0 (not true at all) to 4 (true nearly all the time), with higher scores reflecting higher level of resilience [29]. The CD-RISC 10 showed good to excellent psychometric properties for individuals with SCI and the best combination of reliability, validity and practicality, as compared to the 25-item and 2-item versions [﻿30﻿]. The total sum is converted to a score from 0 to 100 based on the number of valid answers, with a higher score representing higher resilience.

Due to the brief duration of the intervention and short follow-up period, we do not expect to see major changes in life satisfaction, resilience, physical activity and community participation. However, we decided to include these areas as secondary outcomes. The main reason for the latter was to compare our cohort characteristics with those from other studies and prepare for assessing these areas in longitudinal and controlled studies in the future.

### Sample size

The G* Power software package was used to prospectively calculate the adequate sample size for this study (G*Power Team, Germany downloaded from http://www.gpower.hhu.de/en.html). Assuming a two-sided test with alpha = 0.05 and beta = 0.05 (power 0.95), a small effect size of 0.4 and expecting a 20% attrition rate, we would need 101 cases in each country. Effect size of 0.5 for SCIM-III (10% change in total score) has been shown to be a meaningful change [31]. Inter-PEER aims to find changes that have the same or slightly less sensitivity than SCIM-III. We are not interested in finding changes in primary outcomes that have far less sensitivity than SCIM-III, that is an effect size of less than 0.4, for two reasons. First, capturing even smaller effect sizes in self-reported surveys may not be meaningful for community organisations. Second, being able to identify even smaller effect sizes would necessitate a large number of participants, which may not be realistic for some community organisations offering only a few programmes.

### Translation of outcome measures

**Fig. 1:**
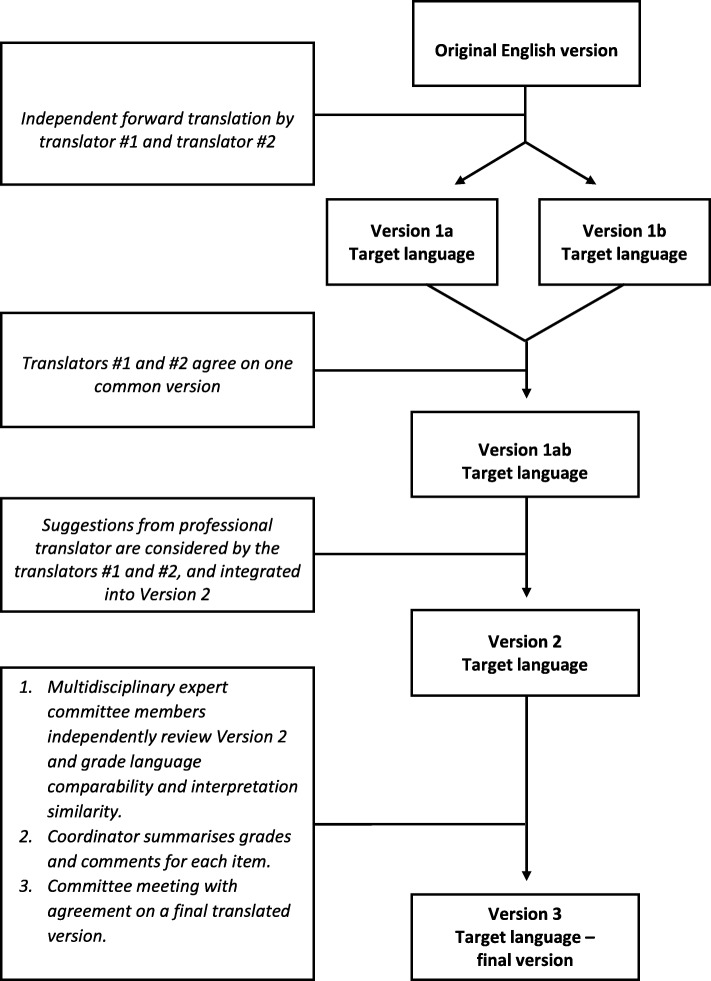
Process for translation of outcome measures in the INTERnational Project for the Evaluation of activE Rehabilitation (inter-PEER)

In each country where the inter-PEER protocol is implemented, a local research team translates all outcome measures that are not available in their native language. To ensure high level of linguistic translation and cultural adaptation, we use a process that is inspired by the work of Fekete et al. [﻿20﻿] and Augutis et al. [32], and is based on previously published guidelines and recommendations [33, 34, 35]. The translation process is described in Fig. 1. The first step involves a forward translation conducted independently by two translators from English into the target language to generate Versions 1a and 1b. One translator is a health professional working in SCI rehabilitation and the other is an experienced peer mentor with SCI - both are native speakers of the target language and with proficiency in the original language (i.e. English). The two translators agree on a synthesized version (version 1ab) derived from the two independent versions (versions 1a and 1b). This version is reviewed by a professional translator who is also a native speaker of the target language. The professional translator gives feedback and suggestions for alterations which are then considered and integrated by the two first translators into Version 2.

Version 2 is then reviewed by a multidisciplinary expert committee. Prior to the committee meeting, each member of the committee is asked to compare and rank Version 2 of the outcome measure as compared to the original English version in terms of language comparability and interpretation similarity. Language comparability (i.e. formal similarity of words, phrases, and sentences) and interpretation (i.e. the degree to which the two versions would engender the same attitude response even if the wording were not the same) are rated on a 7-point scale, similar to that published by Sperber et al. [36]. This step allows for identification of potentially problematic items (those items with low ranking) and retranslate them until all committee members are satisfied with the quality of the translation [36]. The committee members then send the grading of items and their individual comments and suggestions for alterations to the coordinator of the process who synthesizes the comments to determine the differences between the translated (Version 2) and the original version. At the committee meeting, these differences are discussed in detail and the committee agrees on version 3; the final translated version.

The expert committee comprises everybody involved in previous steps, as well as other professionals of a multidisciplinary rehabilitation team, i.e. physicians, physical therapists, occupational therapist, psychologist and peer mentors with SCI. Developers of the original outcome measures have been asked to provide an approval for translating and adapting the measures into the target language. The final translated version of each outcome measure is tested for its psychometric properties. The final translated version is provided to the developers of the original version, and together with the results from the testing it is published as part of a scientific paper.

### Involvement of people with SCI in the project

People with SCI have been involved in all stages of the inter-PEER. The coordinator of many of the AR training programmes in Sweden (Erik Berndtsson; EB) is a peer mentor with SCI. EB has provided continuous advice about the design of this study, and especially on how to integrate the survey better into the schedule of the programmes and on how to increase the response rate. EB, Erika Nilsson (employee at the Spinalis Foundation), the second author (TT) and Robert Jagodziński (employee at the Polish AR organisation), are all persons living with SCI and were involved in the linguistic translation and cultural adaptation of outcome measures in Sweden and Poland, respectively. Participants and peer mentors with SCI at the Swedish AR training programme in 2018 were involved in piloting the data collection processes and provided feedback about the content of the survey as well as about ways to reduce the burden of participation.

### Statistical analysis plan

The assumption of normality will be tested by visually inspecting histograms and by using the Kolmogorov-Smirnov test. *In case of normal distribution, a mixed model* analysis of variance (ANOVA) for repeated measures with auto-regressive covariance structure will be used for variables with three measurements and the paired t-test for variables with two measurements. *In case of non-normal distribution, the* ANOVA non-parametric Friedman test of variance will be performed for variables with *three measurement*s and the *Wilcoxon* signed-rank *test for variables with* two *measurements. To* describe *the magnitude of the difference between different times of measurement, the effect size* (d) will be calculated as the difference between means divided by the standard deviation of the difference. Using Cohen’s criteria [37], an effect size ≥0.20 and < 0.50 will be considered small, ≥ 0.50 and < 0.80 medium, and ≥ 0.80 large.

### Missing data

For outcome measures where the total score is converted to percent based on the number of valid responses (i.e. USER-Participation; WST-Q; CD-RISC 10), we will consider responses only if the respondent has answered two thirds of the items. For outcome measures where the total score is the sum of items (i.e. SCIM-SR; LiSat-11; MSES), we will use the ipsative imputation method if the percentage of missing items for each outcome measure is 20% or less [38]. We will do that by substituting the missing items by the mean of the remaining items within the individual [38]. This approach has been reported as a valid imputation method for a similar type of self-reported questionnaire [38]. All missing data in QEWS and LTPAQ-SCI will be considered and reported as such without any manipulation.

### Lessons learnt from the pilot phase

To ensure the feasibility of the research protocol, we conducted a pilot that included seven participants with diverse spinal cord lesion and sociodemographic characteristics in Sweden. In addition to having the participants complete the evaluation survey and wheelchair skills test, we collected feedback about study procedures through interviews with participants, peer-mentors and programme organisers, as well as through field observations. We identified several problems with the initial version of the protocol: i) the survey was perceived as very lengthy and taking a long time to complete; ii) the use of paper and electronic means to complete the survey was perceived as inconvenient; iii) the data collection was not sufficiently integrated into the schedule of the AR programme, making the participants feel as if they were missing out on other programme activities; iv) some outcome measures (e.g. LTPAQ-SCI), were perceived as difficult to comprehend without guidance from the on-site data collection coordinators, which could result in low quality or missing data; v) the surveys during the programme had a high response rate but the follow-up survey had a high rate of dropouts; vi) some participants raised concerns with confidentiality issues (e.g. due to the rather low incidence of SCI in Sweden, members of the research team may know participants personally); and vii) the need to re-enter data if internet connection was interrupted while completing the survey. All these issues were addressed when developing the updated version of the protocol. The survey was shortened to minimize overlap between questions. The survey was made entirely web based and participants can also continue the survey where they left it if they need to take a break or connection is interrupted. The evaluation survey and wheelchair skills test are completely integrated into the AR programme with the help of the programme coordinator and the board of the AR organisation. The participants are contacted with a personalized e-mail and provided with their results on the wheelchair skills test (i.e. QEWS) to encourage participation in the 3-month follow-up.

### Feedback from the pilot phase

Qualitative feedback from participants in the pilot phase of the study highlighted the importance of the data collection at baseline: “it [the evaluation] made me think why I am here and what I could achieve”. The practical wheelchair skills test was seen as “…a good way to see your progress”. Participants also suggested to “use the findings from the QEWS to inform the focus of wheelchair skills training”. In regard to the follow-up, participants indicated that “follow-up survey and phone call are good ways to be motivated to continue to improve”.

Feedback from peer mentors highlighted the benefits from using QEWS: “It is good to evaluate the effects in an objective, easy and meaningful way”. Also, peer mentors indicated that “results from the wheelchair skills test could be used in the goal setting discussion during the AR programme”. Lastly, the benefits of involving peer mentors and the programme coordinator with follow-up was highlighted by stating that “follow-up assessment is a good way to keep in touch with participants, and identify if there is anything major that they need help with”.

## Discussion

### Dissemination

Findings of the Inter-PEER will be shared with academics and the wider scientific community through peer-reviewed publications, seminars and conference presentations. There will be a series of articles specific for each country and articles based on pooled data. Findings will also be communicated to people with SCI through lay language articles in consumer-oriented magazines and newspapers, as well as on the websites of the community organisations. Procedures and findings will be further disseminated to community organisations through workshops and technical reports.

### Significance

*At an operational level*, findings from the inter-PEER will be utilised to modify the design of existing AR programmes to better serve the needs of future participants. Furthermore, findings may strengthen the case for developing such programmes in countries with limited availability of community rehabilitation. This is aligned with Objective 2 of the WHO Global Disability Action Plan 2014–2021 “*to strengthen and extend community-based rehabilitation*” [39].

*At a research level*, inter-PEER will establish the use of standardised, internationally accepted outcome measures in the AR context, which will encourage organisations offering AR services to evaluate outcomes. Organisations will find it easier to conduct research as they will have a scientific toolkit that they can consider using. The availability of a battery of outcome measures and of standardised research processes will stimulate international collaborations and inform the design of future studies in this area. This protocol and the findings from prospective cohort studies will also inform the design of future controlled and potentially randomised studies that will explore the effectiveness of the AR training programmes. This is aligned with the WHO initiative “Rehabilitation 2030” that has called for action to upscale rehabilitation and highlighted the importance of building research capacity in order to expand the availability of robust evidence for rehabilitation [40].

*At the health system level*, inter-PEER will provide evidence for the role of AR programmes in the continuum of SCI care, i.e. acute care and in-patient rehabilitation, which may have further implications for policy and organisation of health care systems. In turn, this may help organisations that provide AR programmes to integrate better into the health care system, strengthen and streamline referral processes, secure funding and reach more community-dwelling individuals with SCI who could benefit from such services. Better integration does not mean merging with the health care system, but rather the opportunity to establish joint goals and responsibilities, close partnership, high degrees of mutual trust and respect, joint arrangements for streamlining processes including referrals, funding allocation and joint arrangements encompassing strategic and operational issues [41]. This is aligned with Objective 1 of the WHO Global Disability Action Plan 2014–2021 to “*remove barriers and improve access to health services and programmes*” [39].

At an *international level*, inter-PEER can assist with implementing similar evaluations of AR programmes in countries where these programmes are available. The protocol would need to be adapted to suit the processes, needs and resources of the respective organisations and an ethics approval would be needed to be obtained. This could facilitate the implementation of larger and more robust studies by pooling data across countries. This is aligned with Objective 3 of the WHO Global Disability Action Plan 2014–2021 to “*strengthen collection of relevant and internationally comparable data on disability and support research on disability and related services*” [39].

## Supplementary information

**Additional file 1.** The Template for Intervention Description and Replication (TIDieR) for Active Rehabilitation programmes.

## Data Availability

All data are handled and archived according to the General Data Protection Regulation (GDPR) to attain confidentiality and is available from the principal investigator upon reasonable request.

## Abbreviations

ANOVA: Analysis of variance
AR: Active Rehabilitation
CBR: Community-based rehabilitation
CD-RISC 10: 10-item Connor-Davidson Resilience Scale
HADS: Hospital Anxiety and Depression Scale
inter-PEER: INTERnational Project for the Evaluation of activE Rehabilitation
ISCoS: International Spinal Cord Society
LiSat-11: 11-item Life Satisfaction Questionnaire
LTPAQ-SCI: Leisure Time Physical Activity Questionnaire for people with SCI
MSES: Moorong Self-efficacy Scale
QEWS: Queensland Evaluation of Wheelchair Skills
SCI: Spinal cord injury
SCIM-SR: Spinal Cord Independence Measure Self-report
STROBE: Strengthening the Reporting of Observational studies in Epidemiology
TiDieR: Template for Intervention Description and Replication
USER-Participation: Utrecht Scale for Evaluation of Rehabilitation Participation
WHO: World Health Organisation
WST-Q: Wheelchair Skills Test Questionnaire

## References

[1] Dickson A, Ward R, O'Brien G, Allan D, O'Carroll R (2011). Difficulties adjusting to post-discharge life following a spinal cord injury: an interpretative phenomenological analysis. Psychol Health Med.

[2] Wallace MA, Kendall MB (2014). Transitional rehabilitation goals for people with spinal cord injury: looking beyond the hospital walls. Disabil Rehabil.

[3] van Leeuwen CM, Post MW, van Asbeck FW, Bongers-Janssen HM, van der Woude LH, de Groot S (2012). Life satisfaction in people with spinal cord injury during the first five years after discharge from inpatient rehabilitation. Disabil Rehabil.

[4] Boschen KA, Tonack M, Gargaro J (2003). Long-term adjustment and community reintegration following spinal cord injury. Int J Rehabil Res.

[5] Divanoglou A, Tasiemski T, Augutis M, Trok K (2017). Active rehabilitation-a community peer-based approach for persons with spinal cord injury: international utilisation of key elements. Spinal Cord.

[6] Divanoglou A, Georgiou M (2017). Perceived effectiveness and mechanisms of community peer-based programmes for spinal cord injuries-a systematic review of qualitative findings. Spinal Cord.

[7] Chaffey L, Bigby C (2018). Health education by peers with spinal cord injury: a scoping review. J Dev Phys Disabil.

[8] von Elm E, Altman DG, Egger M, Pocock SJ, Gotzsche PC, Vandenbroucke JP (2007). Strengthening the reporting of observational studies in epidemiology (STROBE) statement: guidelines for reporting observational studies. BMJ.

[9] Hoffmann TC, Glasziou PP, Boutron I, Milne R, Perera R, Moher D (2014). Better reporting of interventions: template for intervention description and replication (TIDieR) checklist and guide. BMJ.

[10] Van Stan JH, Dijkers MP, Whyte J, Hart T, Turkstra LS, Zanca JM (2019). The rehabilitation treatment specification system: implications for improvements in research design, reporting, replication, and synthesis. Arch Phys Med Rehabil.

[11] van Langeveld SA, Post MW, van Asbeck FW, Postma K, Ten Dam D, Pons K (2008). Development of a classification of physical, occupational, and sports therapy interventions to document mobility and self-care in spinal cord injury rehabilitation. J Neurol Phys Ther.

[12] Fekete C, Eriks-Hoogland I, Baumberger M, Catz A, Itzkovich M, Luthi H (2013). Development and validation of a self-report version of the spinal cord Independence measure (SCIM III). Spinal Cord.

[13] Gollan EJ, Harvey LA, Simmons J, Adams R, McPhail SM (2015). Development, reliability and validity of the Queensland evaluation of wheelchair skills (QEWS). Spinal Cord.

[14] Kirby R, Rushton P, Smith C, Routhier F, Best KL, Cowan R, et al. The Wheelchair Skills Program Manual Published electronically at Dalhousie University, Halifax, Nova Scotia, Canada.: Dalhousie University; [Available from: https://wheelchairskillsprogram.ca/en/]

[15] Middleton JW, Tate RL, Geraghty TJ (2003). Self-efficacy and spinal cord injury: psychometric properties of a new scale. Rehabil Psychol.

[16] Fugl-Meyer AR, Bränholm I-B, Fugl-Meyer KS (1991). Happiness and domain-specific life satisfaction in adult northern swedes. Clin Rehabil.

[17] Post MW, van der Zee CH, Hennink J, Schafrat CG, Visser-Meily JM, van Berlekom SB (2012). Validity of the Utrecht scale for evaluation of rehabilitation-participation. Disabil Rehabil.

[18] Martin Ginis KA, Phang SH, Latimer AE, Arbour-Nicitopoulos KP (2012). Reliability and validity tests of the leisure time physical activity questionnaire for people with spinal cord injury. Arch Phys Med Rehabil.

[19] Campbell-Sills L, Stein MB (2007). Psychometric analysis and refinement of the Connor-davidson resilience scale (CD-RISC): validation of a 10-item measure of resilience. J Trauma Stress.

[20] Fekete Christine, Post Marcel W.M., Bickenbach Jerome, Middleton James, Prodinger Birgit, Selb Melissa, Stucki Gerold (2017). A Structured Approach to Capture the Lived Experience of Spinal Cord Injury. American Journal of Physical Medicine & Rehabilitation.

[21] Catz A, Itzkovich M (2007). Spinal cord Independence measure: comprehensive ability rating scale for the spinal cord lesion patient. J Rehabil Res Dev.

[22] Kirby RL, Worobey LA, Cowan R, Pedersen JP, Heinemann AW, Dyson-Hudson TA (2016). Wheelchair skills capacity and performance of manual wheelchair users with spinal cord injury. Arch Phys Med Rehabil.

[23] Rushton PW, Kirby RL, Miller WC (2012). Manual wheelchair skills: objective testing versus subjective questionnaire. Arch Phys Med Rehabil.

[24] Middleton JW, Tran Y, Lo C, Craig A (2016). Reexamining the validity and dimensionality of the Moorong self-efficacy scale: improving its clinical utility. Arch Phys Med Rehabil.

[25] Post MW, van Leeuwen CM, van Koppenhagen CF, de Groot S (2012). Validity of the life satisfaction questions, the life satisfaction questionnaire, and the satisfaction with life scale in persons with spinal cord injury. Arch Phys Med Rehabil.

[26] van der Zee CH, Baars-Elsinga A, Visser-Meily JM, Post MW (2013). Responsiveness of two participation measures in an outpatient rehabilitation setting. Scand J Occup Ther.

[27] Martin Ginis KA, Latimer AE, Buchholz AC, Bray SR, Craven BC, Hayes KC (2008). Establishing evidence-based physical activity guidelines: methods for the study of health and activity in people with spinal cord injury (SHAPE SCI). Spinal Cord.

[28] van der Scheer JW, Martin Ginis KA, Ditor DS, Goosey-Tolfrey VL, Hicks AL, West CR (2017). Effects of exercise on fitness and health of adults with spinal cord injury: a systematic review. Neurology..

[29] Connor KM, Davidson JR (2003). Development of a new resilience scale: the Connor-Davidson resilience scale (CD-RISC). Depress Anxiety.

[30] Kuiper H, van Leeuwen CCM, Stolwijk-Swüste JM, Post MWM (2019). Measuring resilience with the Connor–Davidson resilience scale (CD-RISC): which version to choose?. Spinal Cord.

[31] Scivoletto G, Tamburella F, Laurenza L, Molinari M (2013). The spinal cord independence measure: how much change is clinically significant for spinal cord injury subjects. Disabil Rehabil.

[32] Augutis M, Akesson E, Samuelsson K, Antepohl W, Wahman K, editors. A useful process of translation and validation of the ISCoS international basic data sets. 57th International Spinal Cord Society Meeting; 2018; Sydney, Australia. [Available from: https://www.iscos.org.uk/uploads/sitefiles/ISCoS%20Annual%20Meeting%202018/Abstract_Book_Posters_FINAL.pdf]

[33] Biering-Sorensen F, Alexander MS, Burns S, Charlifue S, DeVivo M, Dietz V (2011). Recommendations for translation and reliability testing of international spinal cord injury data sets. Spinal Cord.

[34] Beaton DE, Bombardier C, Guillemin F, Ferraz MB (2000). Guidelines for the process of cross-cultural adaptation of self-report measures. Spine (Phila Pa 1976).

[35] Epstein J, Osborne RH, Elsworth GR, Beaton DE, Guillemin F (2015). Cross-cultural adaptation of the health education impact questionnaire: experimental study showed expert committee, not back-translation, added value. J Clin Epidemiol.

[36] Sperber AD, Devellis RF, Boehlecke B (1994). Cross-cultural translation: methodology and validation. J Cross-Cult Psychol.

[37] Cohen JW (1988). Statistical power analysis for the behavioral sciences.

[38] Imai H, Furukawa TA, Kasahara Y, Ishimoto Y, Kimura Y, Fukutomi E (2014). Ipsative imputation for a 15-item geriatric depression scale in community-dwelling elderly people. Psychogeriatrics..

[39] World Health Organisation (2015). WHO Global Disability Action Plan 2014-2021. Better health for all people with disability.

[40] World Health Organisation (2017). Rehabilitation 2030 - a call for action. Meeting report.

[41] Powell M, Exworthy M, Berney L, Sykes R, Bochel C, Ellison N (2001). Playing the game of partnership. Social Policy Review, 13 : Developments and Debates, 2000-2001.

