# Supplementary material for: INTERnational Project for the Evaluation of “activE Rehabilitation” (inter-PEER) – a protocol for a prospective cohort study of community peer-based training programmes for people with spinal cord injury
Source: BMC Neurol. 2020 Jan 11;20:14. doi: 10.1186/s12883-019-1546-5 (PMC6954505; doi:10.1186/s12883-019-1546-5)
Supplement: Supplementary file 1 — Additional file 1. The Template for Intervention Description and Replication (TIDieR) for Active Rehabilitation programmes. [file 12883_2019_1546_MOESM1_ESM.docx]

Additional file 1. The Template for Intervention Description and Replication (TIDieR) for Active Rehabilitation programmes*

1. Brief name

Active Rehabilitation - community peer-based training programmes.

1. Why

Qualitative research indicates that community peer-based programmes are beneficial for individuals with SCI, and may be the only way for them to interact with others living with similar disability. AR programmes provide face-to-face structured sessions and informal interactions between participants and peer mentors. AR programmes provide opportunities for physical training, to acquire new knowledge and improve behaviour/ attitude. The programmes are led and delivered by trained peer mentors who have a lived experience of SCI. Peer mentors are a credible resource, a living example of what the participants could achieve and a resource for how to achieve it.

1. What material & 4. What procedures

On average, every AR programme has one hour of wheelchair skills training, one hour of ADL training, one hour of strength and fitness training, two hours of sports and recreational activities (e.g., table tennis, swimming, archery, wheelchair basketball and wheelchair rugby, depending on available equipment and facilities) and a half hour of educational sessions per day. Educational sessions may include: a) introduction to living with SCI (e.g., availability of assistive equipment, employment legislation), b) wheelchair adjustments, c) prevention of secondary health conditions (e.g., urinary tract infections, pressure sores) d) bowel management, e) sexuality, fertility and parenting. Informal interactions between participants and peer mentors are typically related to physical independence, practical tips, behaviour and attitude about daily life situations.

*Training* aims at improving accuracy, speed, automaticity of performance, preventing deterioration of normal or optimal performance in transfers, standing, walking, manual wheelchair propulsion, transportation, moving around indoors and outdoors, ashing oneself, caring for body parts, toileting, dressing; eating and drinking.

*Educational sessions* aim at enhancing knowledge (increase amount, improve accuracy) about what is possible to achieve; physiology and anatomy of spinal cord and pathophysiology of spinal cord injury; how to avoid long-term complications; nutrition and physical activity; availability of assistive equipment and how to choose, adjust and use it; adaptive sports and recreational activities in the community; sitting posture; how to train correctly; knowledge about sex, relationships, fertility and parenting; and knowledge about employment legislation.

*At a behavioral level*, the programme aims at improving attitudes towards using a wheelchair; behavior and attitude towards physical appearance and hygiene; motivation to practice and train on a regular basis; attitude towards functional level and limitations; attitutde towards SCI-related conditions e.g. spasticity, pain;

confidence about managing the unexpected; increased self-confidence;

feeling of mastery; attitude towards own ability to take part in society and maintain social life.

There are training ramps for wheelchair training, gym equipment (e.g. free weights and weight machines), rehabilitation equipment (e.g. walking aids, transfer boards, walking belts), sports equipment (e.g., sport wheelchairs).

1. Who provided

*Peer mentors* have a lived experience of SCI and deliver the programme as trainers, educators and living examples. Peer mentors may be volunteers or paid, and some of them also work in a similar role in healthcare institutions. Each community organisation has access to a pool of peer mentors. The programme organiser selects the peer mentors based on the characteristics of enrolled participants.

*Non-disabled assistants* support and help the peer mentors and participants during AR programmes (e.g., practical set up of activities; support participants in transfers, ADL or training situations when necessary). They often have a health professional education background (e.g., studying or working as physiotherapists, occupational therapists, nurses, personal assistants).

*Other participants* with variable level of experience, background and type of injury can also be the source of learning.

Peer mentors and non-disabled assistants attend educational workshops regularly. These workshops include a variety of topics, such as anatomy, prevention of complications, health promotion, theoretical and practical aspects of self-management, and organisational aspects.

More than 80% of the sessions are led by peer mentors. The occasional few sessions that may not be led by a peer mentor are led by other members of the community organisation or external collaborators who have expertise in that specific activity. Specialised healthcare professionals working with SCI-rehabilitation may be involved in such educational sessions. Peer mentors are always present and involved in all sessions. In high-income countries, on average, there are 17 (±9) participants per programme, and the ratio of peer mentor to participants is 1:2 (5). The number of non-disabled assistants in each programme depends on the level and type of assistance needed.

1. How

Face-to-face residential programmes. Structured and the informal sessions are primarily group-based and have some degree of flexibility that allows peer mentors to individualise training and facilitate informal interactions.

*Training* involves repetitive practice, learning by doing, time-limited target (be ready at a specific time), structured practice, practice in own time in semi-supervised environment, feedback, mirroring instructions, demonstrations, provision of strategies, coaching guidance, progression in demands on performance to maintain an optimal level of challenge.

*Education* involves positive re-enforment and encouragement, one-to-one communications, group discussions, cammaraderie, persuasion e.g. „if I can do it – you can also do it“, methods to enhance comprehension and retension.

In addition at the above strategies, structured sessions and informal interactions aiming at *behavioral level* involve enabling strategies („yes, it is possible“, „if he/she can do it – I can do it“), modifying internal representations or drive states that affect propensity to act (modified beliefs and values; enhanced motivation; modified attitudes).

Participants are divided into groups based on level of physical independence, sex, age and previous experience with AR programmes. Grouping of participants are used so that peer mentors can control the intensity, dose and target of intervention for each group and for each participant based on his/her capacity, needs and priorities. Depending on the total number of participants in each programme, group size varies between 4-8 participants. Each group of participants is led by a group of peer mentors with similar type of injury. There is a designated peer mentor for each participant overlooking the progress and facilitating the goal setting and evaluation discussions.

1. Where

AR programmes take place in community settings, such as sports or recreational complexes, hotels and schools. Some activities may take place in natural environments such as the sea, a river and a forest. Training of toilet transfers, showering and dressing take place at the natural time and environment. Wheelchair skills training typically involves custom-based ramps and stairs, and progresses to the natural environment including city centres. Informal interactions take place everywhere, including the bedrooms/ dorms, where peer mentors and participants share rooms.

1. When and how much

Programmes last between 7-15 days.

1. Tailoring

As part of the application for participation in an AR programme, the participants state what they would like to achieve through participating in the programme. A few weeks before the programme starts, all participants are contacted by leaders, asked if transport is arranged, if they have new complications (wounds etc), other goals or thoughts they want answered. During the first day of the programme, there is a goal-setting discussion between the participant, the peer mentor and a non-disabled assistant. In the middle of the programme, there are two goal progress discussions among the peer mentors, the assistants and the programme organisers. At the end of the programme, there is a goal evaluation discussion among the participant, the peer mentor and an assistant. The focus of the sessions is informed by the goal setting discussion in the beginning of the programme, and is negotiated between the participant and the peer mentors throughout the programme.

1. Modifications

Not applicable.

1. How well – Planned

There are 10 fidelity criteria that are based on the ten key elements of AR programme: 1) Peer mentors with a ratio of at least one peer mentor for every five participants and leading a minimum of 80% of all structured sessions; 2) Trained non-disabled assistants; 3) ADL and wheelchair skills training provided by peer mentors on average at least two hours daily; 4) Physical training, sports and therapeutic recreation activities incorporated in the schedule on average at least two hours daily; 5) Formal educational sessions (e.g. on bowel and bladder management; sexual function) on average at least 30 minutes daily; 6) Training environment in the community; 7a) Specific admission criteria (i.e. being free of severe medical complications e.g. pressure ulcer, serious infection; being able to push a manual wheelchair on an even surface; being able to follow instructions; not having problems with concentration, memory and orientation); 7b) At least half of the participants and peer mentors having a SCI; 8) A goal-setting process is part of the programme; 9) Peer mentors having received formal training about AR; 10) Duration of the AR programmes between 7-15 days, and participants attending at least 3/4 of the actual programme duration. At the end of the program, the on-site data collection coordinator completes a form with the fidelity criteria for the specific programme.

1. How well – Actual

Not applicable

*More information about structures, processes and outcomes of community peer-based programmes is provided in previously published articles (5, 6).
